# A Case of Post-COVID-19 Fibrosis Mimicking Thoracic Manifestation of Ankylosing Spondylitis

**DOI:** 10.15388/Amed.2021.29.1.10

**Published:** 2022-03-29

**Authors:** Emrah Doğan, Sabri Serhan Olcay, Tuba Çınar Olcay, Utku Tapan, Ozge Tapan, Fatih Alaşan

**Affiliations:** Muğla Sıtkı Koçman University, Faculty of Medicine, Department of Radiology, Mugla/Turkey; Muğla Sıtkı Koçman University, Faculty of medicine, Department of Pulmonology, Mentese/Mugla/Turkey; Muğla Sıtkı Koçman University, Faculty of medicine, Department of Pulmonology, Mentese/Mugla/Turkey; Muğla Sıtkı Koçman University, Faculty of medicine, Department of Pulmonology, Mentese/Mugla/Turkey; Muğla Sıtkı Koçman University, Faculty of Medicine, Department of Pulmonology, Mugla/Turkey; Muğla Sıtkı Koçman University, Faculty of medicine, Department of Pulmonology, Mentese/Mugla/Turkey

**Keywords:** Ankylosing spondylitis, Post-covid fibrosis, Apical fibrobullous disease, Computed tomography

## Abstract

The most common thoracic manifestation form of ankylosing spondylitis is apical fibrocystic changes. It is also known as apical fibrobullous disease (AFBD). The patient was diagnosed with ankylosing spondylitis before 9 years. He suffered COVID-19 infection and passed an intensive care period. However, post-covid fibrosis (PCF) atypically affected dominantly apical zones. If we had no sequential CT evaluations, our case could be easily confused with AFBD. On CT taken before COVID-19, the lung apex was normal. Thus, it was confirmed that there was no rheumatologic thoracic manifestation in the patient before suffering from COVID-19 pneumonia. PCF created similar changes as AFBD. Our case is the first reported paper on this topic.

## Santrauka.

Dažniausiai krūtinės ankilozinis spondilitas pasireiškia fibrocistiniais pakitimais plaučių viršūnėje. Ši liga taip pat žinoma kaip fibropūslinė plaučių viršūnės liga (angl. *Apical Fibrobullous Disease* (AFBD)). Mūsų pacientas ankiloziniu spondilitu serga 9 metus. Susirgęs COVID-19 infekcija, jis kurį laiką buvo gydomas intensyviosios terapijos skyriuje. Tačiau fibrozė, atsiradusi persirgus COVID-19 (angl. *PCF*), paveikė plaučių viršūnės sritis, o tai nėra įprasta. Jei nebūtume atlikę tęstinių KT tyrimų, mūsų atvejį būtų lengva sumaišyti su AFBD. Prieš susergant COVID-19 atliktoje KT, plaučių viršūnė buvo be pakitimų. Taigi, buvo patvirtinta, kad prieš pacientui susergant COVID-19 sukeltu plaučių uždegimu, reumatologinių simptomų krūtinėje nebuvo. Fibrozė, atsiradusi persirgus COVID-19 (angl. *PCF*), pasireiškė panašiais pakitimais kaip AFBD. Mūsų atvejis yra pirmasis, aprašytas literatūroje.

## Introduction

Post COVID-19 fibrosis (PCF) is one of the new descriptions that entered the medical literature after the pandemic [1,2]. Ankylosing spondylitis (AS) is a seronegative spondyloarthropathy characterized by the fusion of the sacroiliac joints and spine, as well as involvement in large and small joints. It got this name because the pathological process resulted in ankylosis. It is less commonly called Bechterew’s disease or Marie Strümpell’s disease. Apical fibrobullous disease (AFBD), the most common thoracic manifestation pattern of AS, characteristically presents as upper lobe dominant fibrosis accompanied by bullous/emphysematous parenchymal changes. On the contrary, COVID-19 affects predominantly lower lobe and peripheral zones. The upper lobes are an anatomical region not mostly involved by COVID-19. Thus, fibrosis is not expected in this area [3-5]. Herein, we present the first case report of PCF mimicking AFBD in a patient with AS.

## Case report

Our patient was a 53-year-old male security guard. H was diagnosed with AS during examinations because of low back pain in 2012, and he was treated with sulfasalazine 4 tablets 2x2 daily, NSAID in this period. The treatment of AS was continued with the same concept. In the history of patient, there was no symptom or radiological finding in terms of lung involvement of AS. Chest CT taken in December 2020 was completely normal. The patient was admitted to a state hospital in March 2021 due to cough and shortness of breath and the diagnosis of COVID-19 was made with a positive PCR test. The patient was interned in the intensive care unit because of the worsening of his general condition. At this time, the patient’s scores were as follows: Apache score: 21, Glasgow Coma Scale: E1M3V1. He was intubated for 12 days and followed in the intensive care unit for 20 days. Sulfasalazine was discontinued during the intensive care process. The patient was referred to our hospital after intensive care treatment for *Acinetobacter boumanii* having grown in the sputum culture. The general condition of the patient was moderate in the course of the hospital admission. He was conscious and his vital signs were normal, he had shortness of breath with effort. Oxygen saturation was 89%, respiratory sounds were normal. Following respiratory physiotherapy and Tigecycline 40 mg of methylprednisolone IV once daily, the patient was discharged with an oxygen saturation of 96%.

**Figure 1:**
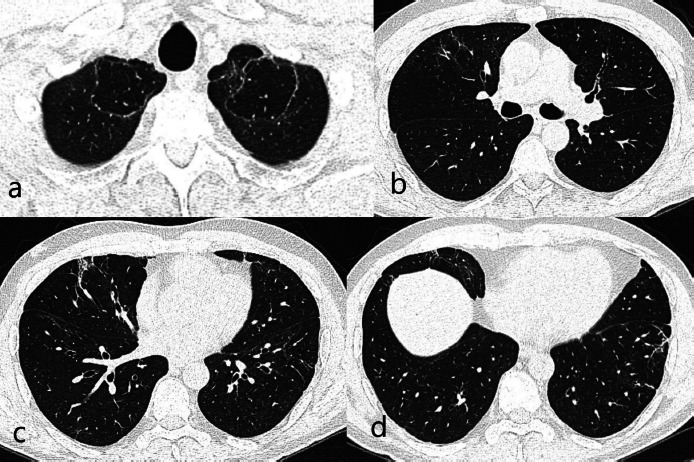
(a). Bilateral apical curvilinear reticular and central bullous changes. (b). Linear reticular changes in the right middle lobe/medial segment. (c). Focal fibrosis areas accompanied with tubular bronchiectasis corresponding middle lobe medial segment inferior part on the right at the level of the inferior pulmonary vein. (d). Reticular fibrotic changes in peripheral areas in the basal segments.

Radiologically, the last CT images showed bullous areas surrounded by linear fibrotic bands at the lung apexes (June 2021). The appearances were partially symmetrical and bilateral. In addition, focal thickenings were observed in the pleura but this appearance was more prominent in middle and lower zones. Subpleural minimal fibrotic changes were present. Mild tubular bronchiectasis at the basal segments was accompanied by fibrosis. However, the findings were not high enough to suggest PCF [Figure 1].

In the CT images (March 2021) taken in a state hospital during intensive care, consolidations and ground-glass opacities (GGO) were seen in the same areas with apical reticular and bullous changes as seen in last CTs, and overlapped with the appearance in the last images.

**Figure 2:**
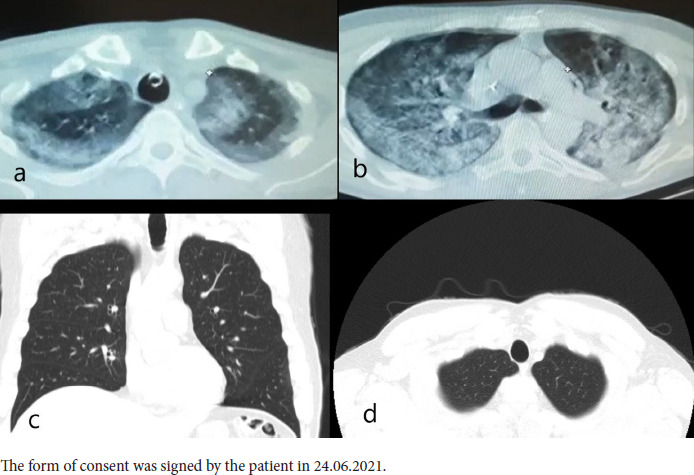
(a). The CT scan performed at the public hospital showed consolidation and GGO areas corresponding anatomically to the same location with apical reticular and bullous changes at the bilateral pulmonary apex. (b). Generalized consolidation and GGO were observed in the axial CT image from carina level. (c). Coronal and (d). Axial CT images before infection with COVID-19 showed that the pulmonary apices were clear.

In the CT images which belong to the period before COVID-19 infection (December 2020), the upper lobes were clean. That is to say, reticular and bullous changes appeared after COVID-19 pneumonia. Thus, the patient got diagnosed with PCF, and AFBD was not considered based on serial radiological evaluation [Figure 2].

## Discussion

It has been revealed that COVID-19 pneumonia leads to a high rate of fibrosis after the pandemic. Post-infectious fibrosis of the COVID-19 has been named PCF [6]. These fibrosis patterns have naturally added to the differential diagnosis with other fibrotic and sequela changes [7]. The lung apex is not one of the predilection zones of COVID-19 infection [8]. Upper zone dominant fibrobullous changes were detected in our patient after infective process.

The development stages of PCF have been roughly described. While pneumonic consolidation regresses, it transforms to GGO, or the GGO may appear without consolidation. During the regression, scattered fibrotic bands may overlap with GGOs. In this case, a picture of GGO along with reticular pattern is formed. Finally, the process terminates with reticular changes in several forms as well as honeycomb changes and bronchiectasis [9,10]. Besides, according to literature, consolidated areas gathered around emphysematous areas have been reported in a patient with British strain. The association of PCF with emphysema or bullous changes is very rare as far as we know [11].

It is also known that COVID-19 pneumonia can lead to rheumatological acute exacerbations [12]. However, our patient got a diagnosis of PCF since there were changes that overlapped with the pneumonia area.

The most important factor enabling us to diagnose in this case was the availability of the followup CT. Radiological follow-up is critical in the evaluation of thoracic manifestation of rheumatological diseases [5]. CT was widely used at the beginning of the COVID-19 pandemic. Quite high rates of sensitivity, specificity and accuracy have been reported. It has even been an alternative method to PCR [13]. However, at the late period CT usage was reduced due to the financial concerns in health management, and with the recognition of the disease and the increase of clinical experience of physicians, PA chest X-ray began to be used more frequently. However, PA chest X-ray is not an examination that can clearly detect the pattern in rheumatological diseases. It does not provide information about low-level progression and regressions. If we do not have radiological CT follow-up in rheumatological patients, PCF cases may confuse even in the presence of X-ray images, as in our patient [3].

This case report has some limitations. There were three different CT scannings during the disease process. The second CT was taken in the local state hospital and was of low quality and there was no detailed information about this time interval.

## Conclusion

In conclusion, PCF can mimic AFBD. Our case is the first reported case on this topic. Chest CT follow-up is important in rheumatologic patients. As in our case, if previous radiological images are present, PCF can be easily diagnosed.
